# Dietary Supplementation with Pomegranate and Onion Affects Lipid and Protein Oxidation in the Breast Meat, Thigh, and Liver, Cellular Stress Protein Responses, and Gene Expression of Liver Enzymes Involved in Protein Synthesis in Broilers

**DOI:** 10.3390/foods12203870

**Published:** 2023-10-22

**Authors:** Soumela Savvidou, Nikolas Panteli, Vassilios Dotas, George Symeon, Dimitrios Galamatis, Ioannis Panitsidis, Eirini Voutsinou, Christina Tatidou, Prafulla Kumar, Efthimia Antonopoulou, Georgios Michailidis, Ilias Giannenas

**Affiliations:** 1 Research Institute of Animal Science, Hellenic Agricultural Organization Demeter, 58100 Giannitsa, Greece; savvidou@elgo.gr (S.S.); gsymeon@elgo.gr (G.S.); 2 Laboratory of Animal Physiology, Department of Zoology, School of Biology, Aristotle University of Thessaloniki, 54124 Thessaloniki, Greece; nkpanteli@bio.auth.gr (N.P.); voutsinou@bio.auth.gr (E.V.); ctatidou@bio.auth.gr (C.T.); eantono@bio.auth.gr (E.A.); 3 Laboratory of Nutrition Physiology & Applied Farm Animal Nutrition, Department of Animal Production, School of Agriculture, Aristotle University of Thessaloniki, 54124 Thessaloniki, Greece; 4 Department of Animal Science, School of Agricultural Sciences, University of Thessaly, 41500 Larissa, Greece; dgalamatis@uth.gr; 5 Laboratory of Nutrition, Faculty of Veterinary Medicine, Aristotle University of Thessaloniki, 54124 Thessaloniki, Greece; panitsid@vet.auth.gr (I.P.); igiannenas@vet.auth.gr (I.G.); 6 Department of Technical Sales and Research, R&D LifeSciences, 8801 Enterprise Blvd, Largo, FL 33773, USA; spcarf@gmail.com; 7 Laboratory of Physiology of Reproduction of Farm Animals, Department of Animal Production, School of Agriculture, Aristotle University of Thessaloniki, 54124 Thessaloniki, Greece; michageo@agro.auth.gr

**Keywords:** *Punica granatum*, *Allium cepa*, cyclodextrin, oxidative stability, cellular proteins, gene expression, breast meat, thigh meat, liver, broilers

## Abstract

The present study examined the effects of dietary supplementation with extracts of pomegranate (*Punica granatum*) and onion (*Allium cepa*), either encapsulated in cyclodextrin (POMALCD group) or in an aqueous (POMALAQ group) form, on breast meat, thigh meat, and liver composition, oxidative stability, cellular signaling pathways, and the gene expression of certain hepatic genes. The results showed that breast and thigh meat contained significantly (*p* < 0.05) higher moisture content in the group with the aqueous extract, compared to the control and POMALCD groups. Moreover, the protein content was significantly (*p* < 0.05) higher in the thigh and liver samples of the treated groups in comparison to the control. The iron-induced challenge deteriorated (*p* < 0.001) the lipid and protein oxidative status of the control group, whereas both supplemented groups showed considerable tolerance in all tissues. The supplementation of pomegranate and onion extracts mitigated or maintained heat shock protein (HSP) levels and elevated (*p* < 0.05) the Bcl-2/Bad ratio in thigh and breast meat, whereas mitogen-activated protein kinase (MAPK) activation was modulated at a lower rate. After normalization to β-actin expression, quantitative real-time PCR analysis revealed a significant (*p* < 0.05) induction in the expression of MTR and MSRB1 genes in the liver of the supplemented groups. No differences were observed for the TAT, SMS, and BHMT genes. In conclusion, dietary mixtures of herbal extracts with pomegranate and onion improved protein and lipid oxidative stability in meat, enhanced the hepatic energy status, and exerted ameliorative effects on stress-related proteins. The encapsulated extract of pomegranate and onion, using cyclodextrin as a carrier, appeared to reduce lipid oxidation to a greater extent than the aqueous extract. In contrast, the aqueous extract exhibited higher total antioxidant capacity (TAC) values and provided better protection against protein carbonyl formation.

## 1. Introduction

According to recent predictions, broiler meat will account for 41% of all meat-derived protein consumption by 2030 [1]. This can be clearly explained by modern consumer preferences for healthy, high-protein, and low-fat poultry products, which are increasing. Simultaneously, the poultry industry’s demand for improved productivity and low production costs necessitates alternative feeding strategies and sources of feedstuff [2]. Animal nutritionists are facing the challenge of increasing productivity and decreasing feed costs through novel alternative feed sources derived from agricultural and food industry byproducts. The phytochemical composition of and the bioactive compounds found in low-cost, phenolic-rich waste products from the vegetable, fruit, and fruit juice processing industries, such as pomegranate peel [3,4,5,6] and onion leaves, have received special attention. They are being proposed as antimicrobial agents and natural growth promoters for broilers.

Pomegranate (*Punica granatum* L.), consumed since ancient times, is currently extensively produced in tropical and subtropical areas, where optimal cultivation climate conditions prevail. Besides its distinct and pleasant organoleptic properties, well-documented beneficial ingredients such as antioxidants, minerals, and vitamins, along with consequent biofunctional properties like antioxidant, anti-inflammatory, antimicrobial, and anticancer activities, have motivated consumer interest in pomegranate. With increasing consumption and the thriving pomegranate juice production industry, larger amounts of waste are being generated. Pomegranate consists of 46% juice, 43% bark, and 11% seeds [7]. Pomegranate peel and seeds, the primary by-products of pomegranate processing (mainly through pressing) into juice, constitute about 54% of the fruit, contributing to waste management problems and environmental degradation. Pomegranate peel (PP) has recently gained attention as a potential material for further valorization [8] and is gradually being recognized for its use in animal nutrition [9,10,11,12]. PP has a high percentage of carbohydrates (59.60%), followed by moisture (5.40–5.95%), protein (4.90–8.97%), ash (3.40–4.22%), fiber (16.30–19.41%), and fat (0.85–1.26%) [13,14]. PP has been found to exert antioxidant, antimicrobial, and anticancer effects in animals and humans, due to its high content of secondary metabolites, primarily phenolic acids (e.g., gallic acid, ellagic acid, and caffeic acid), flavonoids (i.e., flavonols (e.g., catechin, gallocatechin, and epicatechin), anthocyanins and hydrolysable tannins, i.e., ellagitannins (e.g., punicalagin), and gallotannins) [8]. Another pomegranate by-product that has gained particular interest as a natural growth promoter in animal production is pomegranate seeds, containing nearly 24% oil [15] and valuable fatty acids, such as punicic acid [12]. Punicic acid (9-cis, 11-trans, 13-cis, or trichosanic acid) is the prevalent form of the C18:3 class and is reported to have a positive impact on health due to its therapeutic action [10,15].

In an attempt to develop alternative in-feed substitutions for chemical growth promoters and antibiotics used in animal nutrition as health and immunity boosters, the animal feed industry has focused on producing more natural organic substances believed to be less hazardous for both the host and the consumer. Onion (*Allium cepa*), a common plant and a widely used ingredient in cooking worldwide, has been proposed as one of these feed additives [16,17]. Following onion processing, whether in household kitchens or at an industrial level, a vast quantity of by-products/waste is generated, including non-edible aerial or root parts, outer peels and skins, and two outer layers [18]. These by-products may include beneficial substances and phytochemicals. Onion waste is a good source of protein (8.3–15.6% dry matter—dm), ash (4.4–8.6% dm), total dietary fiber (169–750 mg/g dm), and minerals like potassium (11.1–15.9 mg/g), calcium (1.8–16.5 mg/g), magnesium (0.6–1.5 mg/g), iron (0.0196–0.8889 mg/g), zinc (0.0162–0.0538 mg/g), manganese (0.0065–0.0288 μg/g), and selenium (0.00003–0.00093 μg/g). It also contains total phenolics (9.4–52.7 mg gallic acid equivalent (GAE)/g dm), flavonoids (7.0–43.1 mg quercetin equivalent (QE)/g dm), and flavonols (6.19–27 mg/g dm) [19]. Onion peels are a good source of carbohydrates (88.56%) but are low in protein (0.88%), ash (0.39%), and crude fiber (0.15%) [20]. Onion extract contains numerous organic compounds, flavonoids, and phenolic acids, which are responsible for its proven antibacterial, antiviral, anti-parasitic, and antifungal properties, as well as antihypertensive, hypoglycemic, anti-thrombotic, antihyperlipidemic, anti-inflammatory, and antioxidant activities [21].

In experiments conducted with poultry, the inclusion of either pomegranate or onion by-products in their diet has shown positive results regarding host health, production parameters, and meat quality. Dietary PP extract has been found to improve feed utilization, enhance the dietary value of meat, boost the humoral immune response, reduce plasma triglycerides content, and decrease lipid peroxidation in broiler chicken breast meat [4]. Importantly, these effects were observed without affecting liver enzyme biomarkers, cholesterol, low-density lipoprotein (LDL) concentrations, or the levels of gene expression related to immune responses [22]. Rezvani et al. [23] reported that the addition of pomegranate peel extract to the diet of broilers improved the proliferation of favorable bacteria in their ileal and cecal digesta, consequently boosting their immunity.

Although the processing and manufacturing of animal feeds aim to provide nutritious food of high quality and digestibility, deviation from the optimal conditions during the application of several techniques may compromise the sufficiency, bioavailability, and quality of nutrients [24]. For instance, heating may result in protein denaturation and deconformation, while lipid oxidation may occur during extensive drying [24,25]. Subsequently, such alterations in animals’ dietary patterns and habits can shift their nutritional status towards stress [26,27]. Generally, under conditions of nutrient deficiencies and dietary restrictions, AMP-activated protein kinase (AMPK), a critical energy sensor of cellular energy status, is stimulated, to regulate metabolism and maintain energy homeostasis [28,29]. Specifically, upon sensing intracellular metabolic stress, AMPK switches off ATP-consuming anabolic processes (e.g., fatty acid, glycogen, and protein synthesis) and triggers ATP-generating catabolic pathways (e.g., glucose uptake and glycolysis) in an effort to restore the energy balance [28,30]. Moreover, nutrient-induced stress, such as amino acid deprivation, may influence the expression of heat shock proteins (HSPs) [31], a family of evolutionarily conserved proteins constitutively expressed in cells. They serve as molecular chaperones to prevent protein aggregation, premature folding, and imbalances in protein homeostasis [32,33]. Changes in the nutrient content of animals’ diets can also provoke oxidative stress [34,35,36,37], potentially leading to mitogen-activated protein kinase (MAPK) activation [38,39]. In response to numerous stimuli such as growth factors, cytokines, and environmental, dietary, and oxidative stresses, the p38 MAP kinases, the extracellular signal-regulated kinases (ERK), and the c-Jun amino-terminal kinases (JNK) are activated. They regulate miscellaneous cellular functions, including proliferation, differentiation, survival, and development [34,40,41,42,43]. Furthermore, nutrient limitations may trigger apoptotic signaling, which functions as a pivotal regulator–determiner of cell survival or death through the equilibrium between anti-apoptotic proteins (e.g., the B-cell lymphoma protein 2, also known as Bcl-2) and pro-apoptotic proteins (e.g., the Bcl-2 associated death promoter protein, also known as Bad). This process promotes the removal of redundant and damaged cells to maintain homeostasis [40,44,45]. Recently, it has been made clear that the identification of genes, as well as their expression, is involved in biological processes, even interfering with feed efficiency and growth rate [46]. Moreover, herbal feed additives of Indian origin have been shown to be involved in gene expression related to the cycle of protein synthesis and the efficient use of amino acids in broilers [47].

The primary aim of this study was to investigate the impact of a distinctive phytobiotic blend extract that includes pomegranate and onion, provided in two forms (aqueous extract and cyclodextrin-encapsulated), on various aspects related to broiler meat and liver quality. Specifically, we examined oxidative processes, cellular responses at the protein level, and alterations in the gene expression patterns of hepatic enzymes associated with protein synthesis. This investigation was carried out in broiler chickens reared under optimal and healthy conditions.

## 2. Materials and Methods

### 2.1. Animal Experimentation and Sampling

The feeding trial with broiler chickens was conducted in accordance with the local animal husbandry laws for experimentation, and biosecurity measures were implemented based on the Greek legislative framework and the Research Committee of the Aristotle University of Thessaloniki, under the project NR 75042 “Animal Nutrition, Novel Feedstuffs and Environmental Footprint”. The health of the chickens was overseen by a veterinarian. All husbandry, euthanasia, and experimental sampling procedures were carried out in a purpose-built experimental facility at the Research Institute of Animal Science, Hellenic Agricultural Organization Demeter, located in Paralimni (latitude 40.45°, longitude 22.27°), Giannitsa, Greece, during September and October 2021.

A total of 120 one-day-old Ross-308 male chicks, generously supplied by the PINDOS Agricultural Poultry Cooperative Hatchery, were randomly divided into three equal groups, each containing four replicates of ten birds. Each replicate was accommodated in separate floor pens, all of which were equipped with infrared lamps to maintain appropriate heating conditions. Temperature, relative humidity, and lighting conditions were meticulously controlled, adhering to the recommendations of the Aviagen^®^ breeding company. A veterinary surgeon diligently monitored the health of the birds twice daily. Respiratory vaccines were administered to the chicks on the 1st day to guard against Newcastle disease (ND) and infectious bronchitis (IB), along with a subcutaneous vaccination against infectious bursal disease (IBD) at the hatchery. The control feed provided did not contain any antimicrobial or anticoccidial agents.

The control group was given a standard diet, while the POMALAQ and POMALCD groups were supplied with the same diet enriched with an aqueous or cyclodextrin-based additive, both of which contained extracts from pomegranate (*Punica granatum*) and onion (*Allium cepa*) peel. The solid/liquid ratio (S/L) was determined at 1/10 *w/v* for each plant material. The pomegranate and onion leaves were dried and added to the feed; a liter of the aqueous or the cyclodextrin extract contained 200 g of dry material in total. Details on feed composition and the preparation of the plant-based extracts can be found in Vasilopoulos et al. [48]. At the conclusion of the 35-day trial period, all the birds were euthanized, following standard commercial procedures. Two randomly chosen birds from each enclosure were selected, and their feathers were removed. Subsequently, their breast and thigh muscles were extracted from the carcass, weighed, and preserved for future chemical analysis. Liver samples were taken, rapidly frozen in liquid nitrogen, and stored at −80 °C until analysis.

### 2.2. Breast Meat, Thigh Meat, and Liver Composition, Lipid and Protein Oxidation Status, and Total Antioxidant Capacity

The carcasses of the selected birds were initially processed according to the slaughterhouse commercial procedures. Liver tissues were collected from a standardized area, i.e., the dorsal left lobe, thigh meat was sampled from the *Biceps femoris*, and breast meat was obtained from the *Pectoralis major*. Additionally, skeleton muscles were carefully skinned and deboned, and all samples were ground using a domestic mixer–grinder. Fresh 200 g minced meat samples underwent analysis to determine their moisture, protein, fat, and ash content by near infrared spectroscopy using a PerkinElmer DA 7250 (Perten Analyzer Instruments, Hägersten, Sweden) in the transmittance mode, using the reference method 2007.04 for meat and meat products, as described previously [48]. Samples for oxidative investigation were thawed overnight at 4 °C and homogenized using a domestic mixer–chopper. For each breast, thigh, and liver sample, 1 g subsamples were weighed and placed into 50 mL centrifuge tubes. To induce lipid oxidation, a modified version of the method outlined by Kornbrust and Mavis [49] was employed. This process entailed the introduction of 1.0 mL of a solution containing 1.138 mM ferrous sulphate and 0.368 mM ascorbic acid. The incubation was conducted at a temperature of 37 °C for durations of either 30 or 60 min. Subsequent to the incubation, both the iron-induced subsamples and the non-induced subsample were promptly subjected to a malondialdehyde assay to assess the level of lipid oxidation. The assessment of thiobarbituric acid-reactive substances (TBARS) in broiler meat samples followed the method outlined by Ahn and Nam [50], with minor modifications. Briefly, the iron-induced samples were mixed with butylated hydroxyanisole and TBA–trichloroacetic acid solution. The mixtures were vortexed, incubated in boiling water, and then cooled before undergoing centrifugation. The absorbance of each supernatant was measured at 532 nm, and lipid oxidation was expressed as TBARS values, representing nanograms of malondialdehyde (MDA) per gram of meat.

To determine protein carbonyls, we employed the method described by Patsoukis et al. [51] on the same meat samples that underwent iron and ascorbic acid oxidation challenges. In this assay, the formation of carbonyls was identified by their reaction with 2,4-dinitrophenylhydrazine (DNPH), resulting in the conversion of carbonyls into 2,4-dinitrophenylhydrazone (DNP–hydrazone), which was then quantified at a wavelength of 375 nm. The determination of protein carbonyl concentration relied on the molar extinction coefficient of DNPH, which is 22 × 10^3^ M^−1^ cm^−1^.

The same samples were subjected to the determination of total antioxidant capacity using the method outlined by Prieto et al. [52], employing a phosphomolybdate reagent. Tissue extracts (100 μL) were vortexed with 1 mL of the reagent and then incubated in a water bath at 95 °C for 90 min. After cooling, the absorbance was measured at 695 nm using a spectrophotometer. A standard curve was generated using ascorbic acid, and a blank was prepared using the phosphomolybdate reagent without the tissue sample.

### 2.3. Protein Apoptosis

#### SDS–PAGE/Immunoblot Analysis

Cellular responses, including the expression of HSPs, the activation of MAPKs, and the Bcl-2/Bad ratio, were assessed using SDS–PAGE/immunoblot analysis. For the total protein extraction, the frozen breast, liver, and thigh tissues were homogenized in a cold lysis buffer containing protease inhibitors, as described in Antonopoulou et al. [53]. The homogenates were centrifuged (10,000× *g*, 10 min, 4 °C), and the resulting supernatants were collected. The protein concentrations in these supernatants were determined and quantified using the Bio-Rad Protein Assay.

Equivalent amounts of protein (80 μg) from three individuals per diet were separated on 10% (*w/v*) acrylamide and 0.275% (*w/v*) bisacrylamide slab gels and then electrophoretically transferred to a nitrocellulose membrane (0.45 μm, Schleicher & Schuell, Keene, NH, USA). Ponceau staining of the membranes was carried out to assess equal protein loading and transfer efficiency. For the blocking of non-specific binding sites, the membranes were incubated for 30 min with 5% (*w/v*) non-fat milk in Tris buffered saline–Tween (TBST) (20 mM Tris–HCl, 137 mM NaCl, 0.1% (*v/v*) Tween 20, pH 7.5). Subsequently, overnight incubation of the membranes at 4 °C was conducted with the following primary antibodies: monoclonal rabbit anti-heat shock protein, 60 kDa (Cat. No. 12165, Cell Signaling, Beverly, MA, USA), polyclonal rabbit anti-heat shock protein, 70 kDa (Cat. No. 4872, Cell Signaling, Beverly, MA, USA), polyclonal rabbit anti-heat shock protein, 90 kDa (Cat. No. 4874, Cell Signaling, Beverly, MA, USA), monoclonal rabbit anti-phospho-p44/42 MAPK (Thr202/Tyr204) (Cat. No. 4370, Cell Signaling, Beverly, MA, USA), monoclonal rabbit anti-p44/42 MAPK (Cat. No. 4695, Cell Signaling, Beverly, MA, USA), polyclonal rabbit anti-phospho-p38 MAPK (Thr180/Tyr182) (Cat. No. 9211, Cell Signaling, Beverly, MA, USA), polyclonal rabbit anti-p38 MAPK (Cat. No. 9212, Cell Signaling, Beverly, MA, USA), monoclonal rabbit anti-phospho-AMPK (Cat. No. 2535, Cell Signaling, Beverly, MA, USA), monoclonal rabbit anti-AMPK (Cat. No. 5831, Cell Signaling, Beverly, MA, USA), monoclonal rabbit anti-Bcl2 (Cat. No. 3498, Cell Signaling, Beverly, MA, USA), and polyclonal rabbit anti-Bad (Cat. No. 9292, Cell Signaling, Beverly, MA, USA). Afterwards, blots were subjected to incubation for 1 h with horseradish peroxidase (HRP)-conjugated secondary antibody (anti-rabbit IgG, HRP-linked Antibody (Cat. No. 7074, Cell Signaling, Beverly, MA, USA)). The blots were thoroughly rinsed in TBST (3 times, 5 min each) before and after the incubation with both primary and secondary antibodies. Antibody binding was visualized as a band through the enhanced chemiluminescence (ECL) detection system (Cat. No. 7003, Cell Signaling, Beverly, MA, USA), with exposure to Fuji Medical X-ray films. Bands were quantified by densitometric analysis using Image Studio Lite (LI-COR Biosciences, Lincoln, NE, USA).

### 2.4. Enzyme Gene Expression of Amino Acids in Liver Samples

For the validation of the microarray results, a total of six liver-specific genes were chosen and subjected to quantitative real-time PCR (RT-PCR) (Table 1). These selected genes included methionine synthase (MTR), tyrosine aminotransferase (TAT), spermine synthase (SMS), methionine sulfoxide reductase B1 (MSRB1), betaine homocysteine S-methyltransferase (BHMT), and β-actin (control). These genes are pivotal for critical cellular functions and play significant roles in various metabolic processes involving amino acids synthesis, such as methionine and tyrosine. As a result, they hold a crucial position in influencing feed efficiency, growth rate, and protein oxidative status.

#### RNA Isolation, RT-PCR Analysis, and Quantitative Real-Time PCR Analysis

Total RNA was extracted from chicken liver samples. PCR amplification of genes was carried out using the primers and conditions specified in the provided table. RT-PCR analysis and quantitative real-time PCR analysis were performed, as previously described by Pfaffl and Michailidis et al. [54,55].

### 2.5. Statistical Analysis of Meat Oxidation, Liver Gene Expression, and Protein Apoptosis Activation

The study was designed using a randomized complete block design (RCB), with the experimental unit being the replication (pen). The significance of differences in lipid oxidation, protein carbonyl levels, changes in MAPKs’ activation, HSPs, Bcl-2, and Bad expression in response to dietary treatments was assessed at the 5% significance level using a one-way analysis of variance (ANOVA). Subsequent post hoc comparisons were conducted using Tukey’s test. The statistical analyses were performed using the SPSS program (SPSS Statistics 25). The expression of the selected genes was assessed using a one-way ANOVA to evaluate the potential significant effects of the different groups. In the case of statistical significance, the differences between the mean values of specific groups were determined using Duncan’s new multiple range test. Throughout all analyses, differences were considered significant at *p* < 0.05.

## 3. Results

### 3.1. Breast, Thigh, and Liver Composition and Oxidative Status

**Figure 1:**
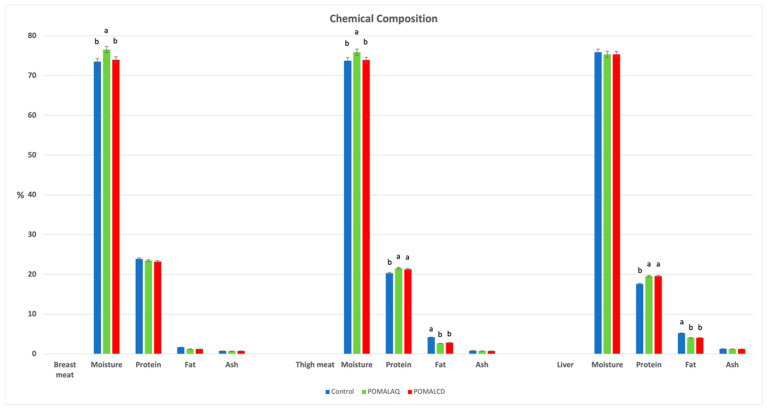
Effect of dietary supplementation with pomegranate and onion on broiler chicken breast, thigh, and liver composition. Control, basal diet; POMALAQ, diet supplemented with aqueous pomegranate and onion extract at 0.1% per kg of DM; POMALCD, diet supplemented with pomegranate and onion in cyclodextrin at 0.1% per kg of DM. ^a,b^ Bars in the same attribute with no common letter differ significantly (*p* < 0.05).

The moisture levels in both breast and thigh meat significantly increased in the POMALAQ group compared to both the control and the POMALCD group. Furthermore, the protein content in the thigh samples of the treated groups was significantly higher in comparison to the control group. In contrast, the fat content in the thigh meat significantly decreased in both treated groups when compared to the control group (Figure 1). Similar trends were observed in liver samples, where significantly lower fat content was evident in the treated groups as opposed to the control group. Additionally, the protein content in the livers of both supplemented groups exceeded that of the control group (Figure 1).

Significant differences in terms of TBARS levels in breast, thigh and liver tissues, after the incubation of samples for either 30 or 60 min with iron, were evident among the treatments (Table 2). Notably, the POMALCD group exhibited the lowest TBARS values at 30 min, and both groups that received the supplemented diets showed a reduction in lipid oxidation compared to the control group. Conversely, the control group displayed the highest TBARS values, suggesting elevated levels of lipid oxidation in the breast and thigh meat, as well as in the liver samples. These observed differences held statistical significance (*p* < 0.001). Similar trends were observed in thigh meat; the POMALCD group showed the lowest TBARS values after iron oxidation at both the 30 and 60 min intervals. In contrast, the control group exhibited significantly higher TBARS values (*p* < 0.001) when contrasted with the treated groups.

Significant differences were observed in terms of protein carbonyls—indicators of protein oxidation—in both supplemented groups for breast, thigh, and liver samples among the treatment groups (Table 2). Remarkably, the POMALAQ group displayed the lowest protein carbonyl levels, signifying reduced protein oxidation in comparison to the control and POMALCD groups, which displayed intermediate values. Conversely, the control group presented the highest protein carbonyl levels, implying an elevated degree of protein oxidation within the meat, and therefore a reduced protective capability. These differences were statistically significant (*p* < 0.001). The TAC values also presented a similar pattern in breast, thigh, and liver samples (Table 2). The lowest total antioxidant capacity was presented in the control group, whereas POMALCD presented a higher (*p* < 0.001) antioxidant capacity than the control group, but a lower (*p* < 0.001) antioxidant capacity than the POMALAQ group.

### 3.2. Cellular Stress Response (Heat Shock Response, MAPK Pathways, and Apoptosis)

**Figure 2:**
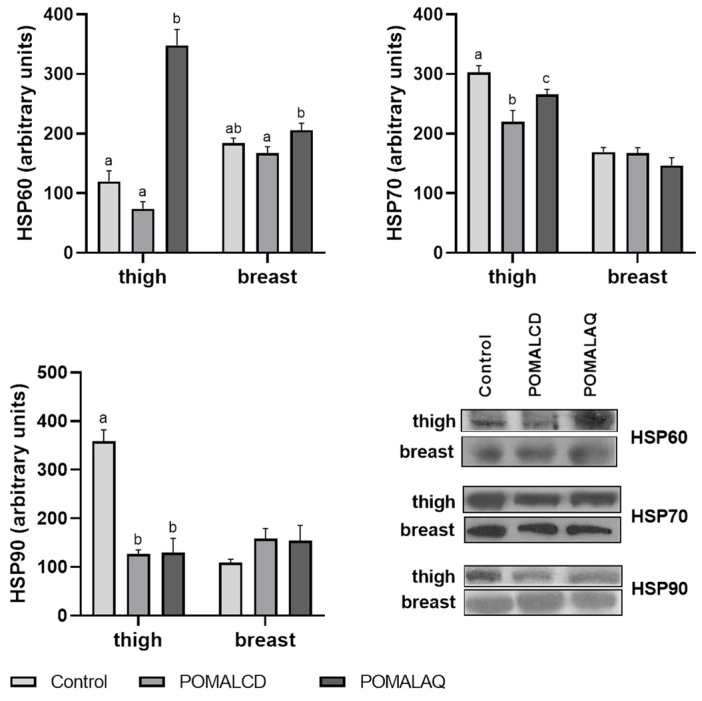
Expression levels of heat shock proteins (HSP60, HSP70, and HSP90) in the thigh and breast tissue of broiler chickens in response to the three dietary treatments, i.e., control, basal diet; POMALCD, diet supplemented with pomegranate and onion in cyclodextrin at 0.1% per kg of DM; or POMALAQ, diet supplemented with aqueous pomegranate and onion extract at 0.1% per kg of DM. Representative protein bands are depicted in the bottom right panel. Data represent means ± SD; n = three biological replicates. Different letters (a–c) indicate significant (i.e., *p* < 0.05) differences among the dietary treatments.

The expression patterns of HSPs in chicken thigh and breast are depicted in Figure 2. For HSP60, there were no significant changes observed in either tissue, when comparing the POMALCD diet to the control. However, the POMALAQ diet resulted in a significant (*p* < 0.05) HSP60 induction in the thigh, whereas no alteration in the expression was observed in the breast, compared to the control. In contrast, HSP70 and HSP90 displayed a similar expression pattern in response to the diets. Specifically, both diets led to a significant (*p* < 0.05) reduction in the levels of HSP70 and HSP90 in the thigh. However, there were no observable changes (*p* > 0.05) in the breast tissue.

**Figure 3:**
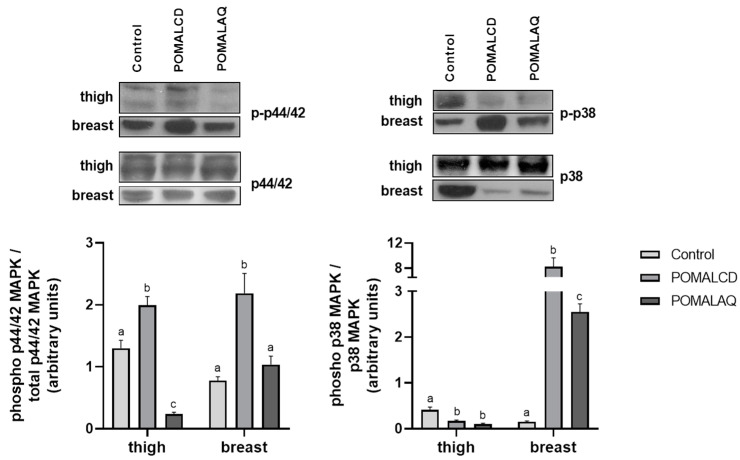
Activation of p38 and p44/42 mitogen-activated protein kinases (MAPKs) in the thigh and breast of broiler chickens in response to the three dietary treatments, i.e., control, basal diet; POMALCD, diet supplemented with pomegranate and onion in cyclodextrin at 0.1% per kg of DM; or POMALAQ, diet supplemented with aqueous pomegranate and onion extract at 0.1% per kg of DM. Representative protein bands are depicted in the top panels. Data represent means ± SD; n = three biological replicates. Different letters (a–c) indicate significant (i.e., *p* < 0.05) differences among the dietary treatments.

Τhe activation of p38 MAPK showed contrasting patterns in the analyzed tissues, as depicted in Figure 3. Specifically, both diets resulted in significantly (*p* < 0.05) reduced p38 MAPK activation in the thigh compared to the control, while a significant activation was evident in the breast of chickens who were fed the two experimental diets. In contrast, the POMALCD diet was linked to a significant increase in p44/42 MAPK phosphorylation in both tissues. On the other hand, chickens fed the POMALAQ diet exhibited a significant reduction in the thigh and a non-significant alteration in the breast, in comparison to the control.

**Figure 4:**
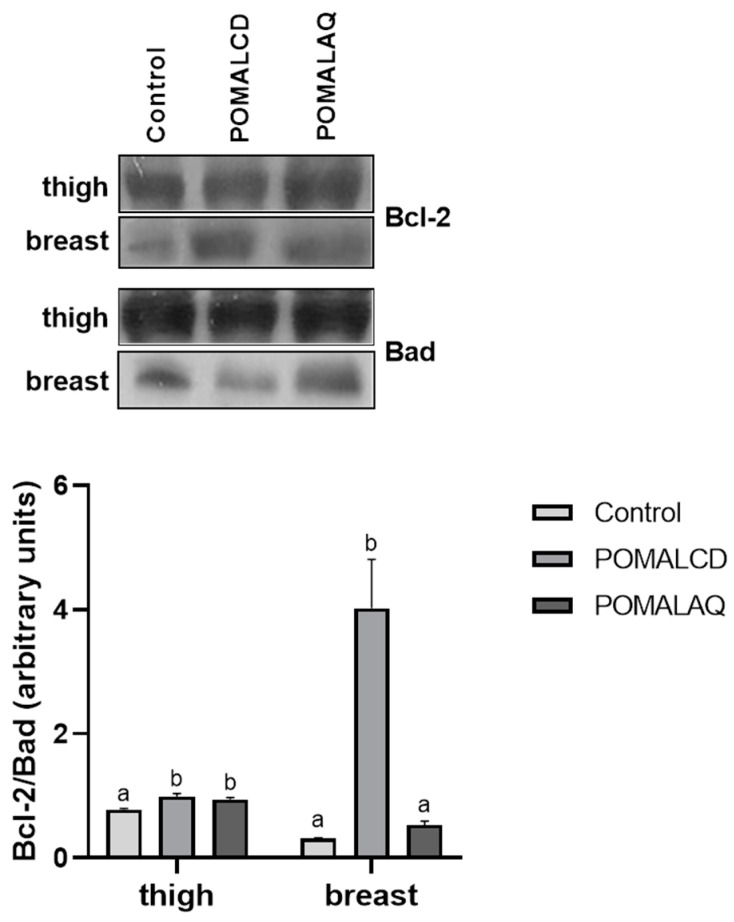
Expression of the apoptotic indicator Bcl-2/Bad ratio in the thigh and breast of broiler chickens in response to the three dietary treatments, i.e., control, basal diet; POMALCD, diet supplemented with pomegranate and onion in cyclodextrin at 0.1% per kg of DM; or POMALAQ, diet supplemented with aqueous pomegranate and onion extract at 0.1% per kg of DM. Representative protein bands are depicted in the top panel. Data represent means ± SD; n = three biological replicates. Different letters (a, b) indicate significant (i.e., *p* < 0.05) differences among the dietary treatments.

The effects of the POMALCD and POMALAQ diets on the apoptotic machinery in chicken thigh and breast are presented in Figure 4. Both diets led to a significant (*p* < 0.05) increase in the Bcl-2/Bad ratio in the thigh, while in the breast of chicken fed the POMALCD diet, an up to ~thirteen-fold increase in the ratio was apparent, compared to the control. However, a non-significant change in the Bcl-2/Bad ratio was observed in the breast in response to the POMALAQ diet.

**Figure 5:**
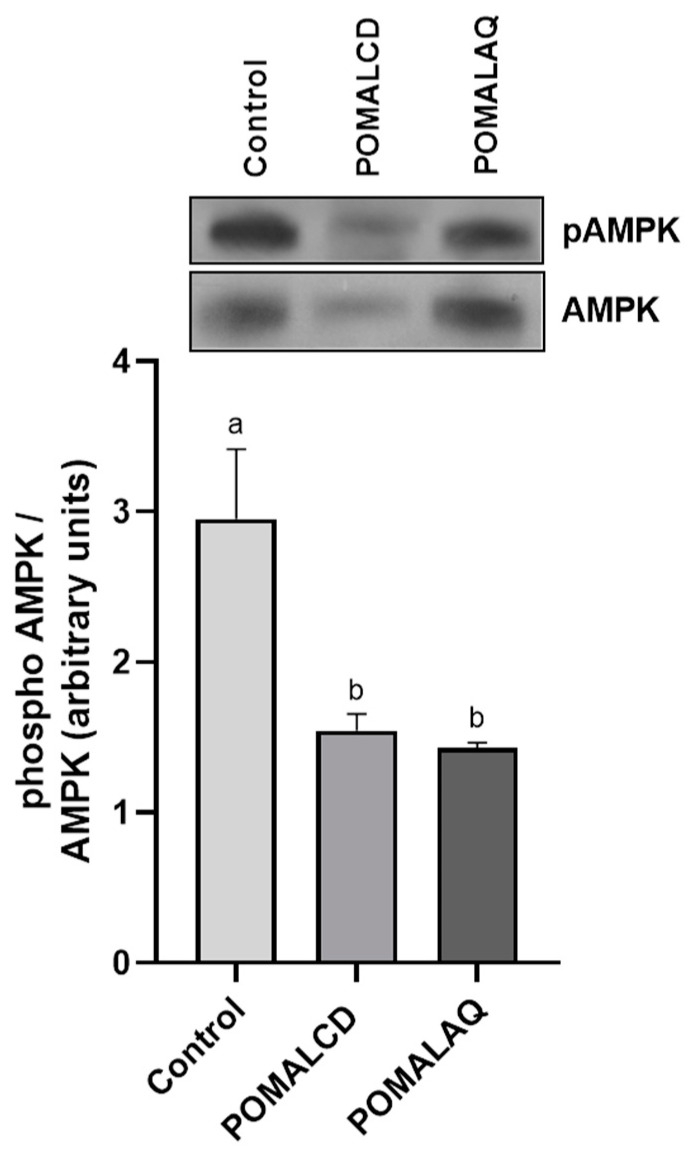
Activation of AMP-activated protein kinase (AMPK) in the thigh and breast of broiler chickens in response to the three dietary treatments, i.e., control, basal diet; POMALCD, diet supplemented with pomegranate and onion in cyclodextrin at 0.1% per kg of DM; or POMALAQ, diet supplemented with aqueous pomegranate and onion extract at 0.1% per kg of DM. Representative protein bands are depicted in the top panel. Data represent means ± SD; n = three biological replicates. Different letters (a, b) indicate significant (i.e., *p* < 0.05) differences among the dietary treatments.

To assess the impact of the pomegranate and onion extract on energy status, the activation of AMPK was examined in the liver, a pivotal organ in the metabolic adaptation of broiler chickens. Remarkably, both the POMALCD and POMALAQ diets led to a significant (*p* < 0.05) reduction in the P-AMPK/AMPK ratio in the liver when compared to the control (Figure 5).

### 3.3. Changes in Gene Expression

**Figure 6:**
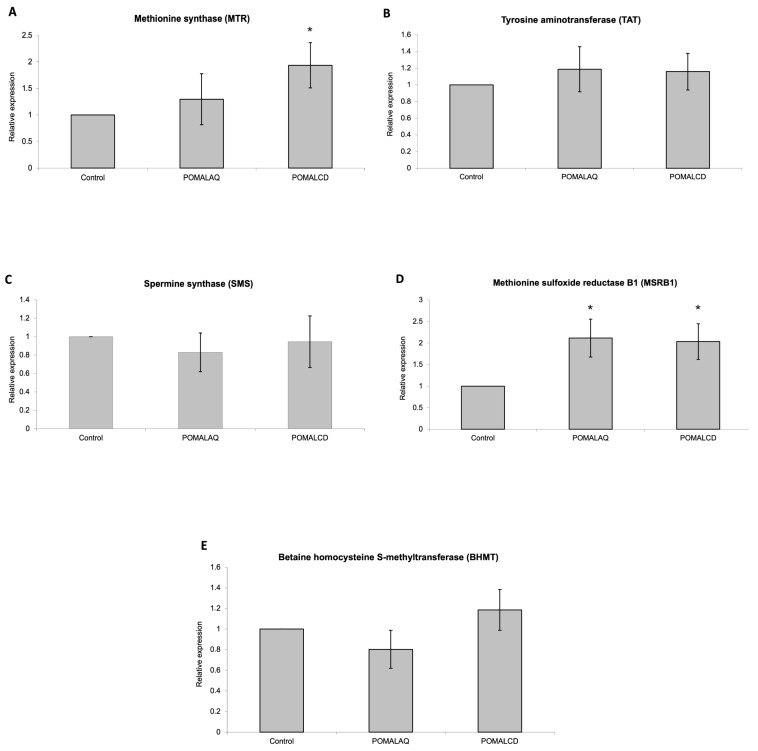
Changes in the expression of MTR (**A**), TAT (**B**), SMS (**C**), MSRB1 (**D**), and BHMT (**E**) genes in the chicken liver, following the three different dietary treatments, i.e., control, basal diet; POMALCD, diet supplemented with pomegranate and onion in cyclodextrin at 0.1% per kg of DM; or POMALAQ, diet supplemented with aqueous pomegranate and onion extract at 0.1% per kg of DM. The mRNA expression levels were examined using quantitative real-time PCR analysis. Values represent the mean ± SEM (n = 12). Asterisk indicates that the differences in the expression levels are statistically significant (*p* < 0.05). (X-axis: different dietary treatments, Y-axis: relative gene expression (fold change)).

After normalization to β-actin expression, quantitative real-time PCR analysis revealed a significant induction (*p* < 0.05) in the expression of MTR and MSRB1 genes in the liver of the POMALCD and POMALAQ groups (Figure 6). The greatest induction was observed for MTR in the liver of the POMALCD group. No differences (*p* > 0.05) were observed for the TAT, SMS, and BHMT genes.

## 4. Discussion and Conclusions

Pomegranate peel extract, derived from the outer layer of the fruit, is rich in bioactive compounds such as phenolic compounds, flavonoids, and tannins, which possess antioxidant, anti-inflammatory, and antimicrobial properties. Dietary inclusion of pomegranate peel extract has shown potential benefits for broiler performance, improving growth performance, feed efficiency, and nutrient utilization [56]. Additionally, it has been found to affect the expression levels of genes related to antioxidant factors and innate immunity, suggesting its potential role in enhancing broiler health [57].

Allium extract, derived from onion, contains various bioactive compounds including flavonoids, organosulfur compounds, and polyphenols, which possess antioxidant, antimicrobial, and immunomodulatory properties. Studies have demonstrated that including onion extract in broiler diets can positively influence broiler performance, resulting in improved body weight gain, feed intake, feed efficiency, modulation of gene expression, and enhanced nutrient digestibility [58].

In our study, we investigated the effects of including diets containing pomegranate and onion extracts in chicken nutrition. Our findings revealed diverse effects on cellular processes and metabolic pathways, including changes in the expression of HSPs, activation of MAPK pathways, modulation of apoptotic machinery, and alterations in energy metabolism. Tissue-specific responses and variations were observed to depend on the specific extracts and their forms.

Regardless of the administered form, dietary supplementation of pomegranate and onion extracts was mainly accompanied by the reduction or maintenance of HSP levels in broilers’ thigh and breast, thereby suggesting the potential nutrient efficacy of pomegranate and onion extract in terms of nutrient stress. Numerous studies have indicated that pomegranate fruit and peel encompass essential bioactive compounds such as flavonoids and hydrolyzable tannins, and exhibit antioxidant, antimicrobial, and anti-inflammatory activity [59,60,61]. Dietary treatment with pomegranate emulsion has been reported to inhibit elevated HSP70 and HSP90 levels and lessen stress induction during diethylnitrosamine (DENA)-evoked hepatocarcinogenesis in rats [62]. Furthermore, pomegranate juice has been described as an oxidative stress mitigator and has been shown to decrease HSP90 expression both in vitro in hypoxic trophoblasts and in vivo in the placentas of pregnant women [63]. Similar to pomegranate, *Allium* species also flaunt antibacterial, anticancer, and antioxidant potential, which are related to the high organosulfur compounds content [64]. For instance, thiosulfinates, such as allicin, contained in *Allium* extracts suppress the growth of numerous bacterial species [65]. Concerning chickens, Enoka et al. [66] demonstrated that extracts from garlic (*Allium sativum*) and onion (*Allium cepa* L.) ameliorate the activity of the antioxidant enzyme catalase in thigh and breast muscle. Moreover, *A. sativum* combined with *Lactobacillus acidophilus* have been reported to enhance the activity of superoxide dismutase in broiler chicken [67]. Despite the lack of research regarding the effects of pomegranate and/or onion on HSP expression in broiler chicken, the present results in conjunction with the above-mentioned findings in the literature may imply that both diets are nutritionally adequate in order to deter subsequent nutrient and oxidative stress induction. As previously mentioned, HSPs are pivotally implicated in the cellular stress response via protein stabilization and the refolding of denatured proteins [32]. Thereby, during oxidative stress due to nutrient deficiency, levels of HSPs may be upregulated to protect protein structure and folding, as has been previously demonstrated in chicken liver in response to selenium deficiency [68].

Regarding MAPKs, differences in activation were evident herein between the cyclodextrin encapsulation and the aqueous form. The differential MAPK activation among the two aforementioned diets may be attributed to the nutritional quality of the phytobiotic mixture entering the gastrointestinal tract, considering that encapsulation ensures the protection of pomegranate and onion extracts from reactivity-induced degradation [69]. The aqueous extract suppressed the activation of both p38 and p44/42 MAPKs in the thigh. The inhibitory effect of pomegranate on MAPK phosphorylation has been previously reported in several studies. Specifically, Wei et al. [70] suggested a protective role of pomegranate peel extract against the carbon tetrachloride (CCl4)-induced fibrosis in rat liver, partially via a reduction in p38 MAPK phosphorylation. Similarly, Khan et al. [71] observed that pomegranate fruit extract decreased the phosphorylation of p38, JNK, and p44/42 MAPKs, which were significantly induced in mouse skin following exposure to solar ultraviolet B (UVB) radiation. Pretreatment with pomegranate flower ethanol extract has also been shown to act as an anti-inflammatory on LPS-stimulated macrophages by suppressing MAPK pathways (p44/42, JNK and p38) [72]. On the contrary, *A. cepa* extract has been reported to facilitate a neuroprotective mechanism against the L-buthionine-S, R-sulfoximine (BSO)-induced oxidative stress in neuronal cells, involving the inactivation of protein kinase C-ε through the phosphorylation of p44/42 MAPK [73]. In addition, the latter study indicated that p44/42 MAPK activation may contribute to the neuroprotective effects of *A. cepa* extract, via the inhibition of p38 MAPK phosphorylation and reactive oxygen species (ROS) production. In agreement with the aforementioned observation, the extract of pomegranate and onion encapsulated in cyclodextrin led to p44/42 MAPK activation and to p38 MAPK suppression in the thigh. Similarly, diallyl disulfide, an organosulfur compound from *A. sativum*, has been shown to activate p44/42-MAPK in human non-small cell lung carcinoma H1299 cells, which may indicate an involvement in the regulation of induced apoptosis, thereby highlighting the anticancer properties of *A. sativum* [74]. Contrary to the thigh, p44/42 MAPK activation in broiler breast meat occurred alongside an increase in the p38 MAPK phosphorylated levels, thus underlying the tissue specificity and susceptibility to dietary changes. Despite the fact that their activation may indicate stress induction in the breast, MAPKs are also components of several multifunctional signaling pathways in cell physiology [41]. Considering that supplementation of pomegranate and onion extract, both encapsulated in cyclodextrin or in an aqueous form, exerted anti-apoptotic effects in the two examined tissues, as indicated by the elevated Bcl-2/Bad ratio, MAPKs activation in the breast may suggest involvement in the transcriptional and post-translational regulation of anti-apoptotic proteins [75]. For instance, JNK-MAPK has been shown to be implicated in apoptosis inhibition via Bad phosphorylation at Thr201, which results in the lessening of its association with the anti-apoptotic Bcl-xL, thus suppressing the pro-apoptotic activity of Bad [76]. Consistent with the present results, oral pomegranate juice supplementation in methotrexate-injected rats led to increased cell proliferation and decreased apoptosis. These effects correlated with an increased p44/42 level and a decreased caspase 3 level, respectively [77]. Thereby, in addition to the contribution in apoptosis inhibition, enhanced p44/42 MAPK phosphorylation may also serve a proliferative function in breast cells. The latter is also supported by the suppression of AMPK activation, observed herein in the liver of broiler chickens, following dietary supplementation with the extract of pomegranate and onion. Functionally, AMPK inactivation suggests that both dietary supplementations enhanced the energy status of broiler chickens and stimulated energy-consuming anabolic processes, including protein synthesis through the upregulation of the target rapamycin (mTOR) signaling pathway, in order to facilitate cell growth and proliferation [30,78]. Pomegranate seed oil has been previously in vitro demonstrated to stimulate the proliferation of human epidermal keratinocytes [79]. Hence, pomegranate and onion extract may exert ameliorative effects regarding growth in breast muscle. Nevertheless, further research is required to clarify whether the state of low AMPK activation is indicative of improved growth or a metabolic disorder onset [80].

Protein synthesis in chickens is an extremely important metabolic process for growth performance and this requirement of feed is of utmost importance to further economize broiler production. Moreover, this process is important for the oxidative status of the bird organism. Despite numerous investigations into the impact of nutrient-deficient diets on chicken growth, the research on liver gene expression related to enzymes involved in methionine cycle synthesis remains notably lacking, both in farm animals and poultry. In a previous study [47], it was shown that specific extracts of Indian herbs such as *Boerhavia diffusa*, *Azadirachta indica*, *Vigna mungo*, and *Trigonella foenum-graecum* can affect gene expression and modulate the growth performance of chickens, even when they are given diets with limited quantities of methionine. It is possible that animal feed containing plant extracts rich in active phenolic ingredients possesses the capability for metabolic, physiologic, and growth promoting properties, resulting in a decrease in humoral and cellular responses and associated gene expressions in broiler chickens. Analyzing gene expression is essential for identifying specific genes and understanding the degree to which they participate in a biological process.

A deficiency in methionine within the diet has been documented to result in changes in the expression levels of multiple genes and the activity of enzymes involved in methionine, like methionine oxidases [81]. Hepatic enzymes have the capacity to regulate the transcription levels of numerous genes that are involved in essential processes related to amino acid biosynthesis and recycling. These include pivotal enzymes like methionine synthase, tyrosine aminotransferase, spermine synthase, methionine sulfoxide reductase B1, and betaine homocysteine S-methyltransferase. Amino acids also serve as influential factors in modulating signaling pathways that govern metabolism and cellular functions [82]. Therefore, gaining insights into the molecular mechanisms that govern the expression of genes related to amino acid metabolism could provide valuable information for optimizing dietary requirements [83]. It is clear that diet supplementation with extracts from aromatic plants may interfere with the gene expression of amino acid in the synthesis of methionine, and, as a consequence, with protein synthesis [47]. Chickens’ diets deficient in methionine have been shown to upregulate the gene expression related to the CAT1 amino acid transporter in large skeletal muscles [83]. Interestingly, CAT1 can stimulate ornithine synthesis over nitric oxide synthesis from arginine. A spare effect on arginine in broilers may accelerate growth performance, as it is the first limiting factor in broiler meat production. The biosynthesis of amino acids such as arginine, ornithine, spermidine, and spermine is associated with essential metabolic pathway of protein biosynthesis.

Chicken meat is prone to lipid peroxidation, as it contains higher levels of unsaturated fats compared to beef or pork meat. Lipid oxidation of poultry meat and fat is unfavorable as it decreases nutritional value, organoleptic characteristics, shelf life, and consumer acceptability of meat and meat products [3]. In order to avoid such conditions, an effective solution seems to be dietary manipulation by including natural antioxidants in broiler diets. Natural antioxidants derived from inexpensive and phenolic-rich waste products, such as pomegranate, have been tested in several experiments, demonstrating their antioxidant activity in broilers [4,5,6,84,85,86,87]. For instance, Sharifian et al. [84] reported that supplementation of pomegranate peel extract in broiler diets preserves the quality of meat during storage. Similarly, Rafiei and Khajali [87] suggested that dietary inclusion of up to 300 mg/kg of pomegranate peel extract improves total phenolic content and antioxidant activity in the breast meat of broiler chickens.

A commonly applied technique in the food processing sector to enhance the stability of food compounds and safeguard them against losses and chemical changes is microencapsulation. Microencapsulation is a novel technique that affirms the stability of the product, gastric protection, delivery, activation, and absorption at the intestinal level, as well as resistance through the pelleting process or the storage of the compound feed. Recently, the microencapsulation process has also found its way into the feed and broiler industry [48], aiming to facilitate inclusion into solid products and offering the potential for more manageable delivery and controlled release during consumption [88].

Regarding lipid and protein peroxidation, the findings of the study demonstrate a significant reduction in lipid and protein oxidation in both breast and thigh meat samples due to the inclusion of pomegranate and onion extract in the diet, under stress oxidative conditions due to the presence of iron and ascorbic acid. Specifically, the POMALAQ group displayed notably lower TBARS values, indicating decreased lipid oxidation compared to the control and POMALCD groups. These results align with previous research, such as the study conducted by Gungor et al. [89], which also reported a decrease in MDA levels in the treated groups with pomegranate supplementation. This outcome can be attributed to the high content of phenolic compounds present in pomegranate and onion, which are well-known for their antioxidant effects.

Similarly, the POMALAQ group demonstrated the lowest levels of protein carbonylation, indicating a reduction in protein oxidation compared to the other groups. This finding supports the notion that pomegranate and onion extracts possess powerful antioxidative properties, effectively inhibiting the oxidative processes responsible for lipid and protein degradation in meat products. These results align with a study conducted by Vasilopoulos et al. [48], which also observed a positive effect of combining pomegranate and onion extracts on protein carbonylation.

In summary, the findings of the present study highlight the efficacy of incorporating pomegranate and onion extract into the diet in effectively mitigating lipid oxidation (as evidenced by reduced TBARS values) and protein oxidation (as indicated by lower protein carbonyl levels). This approach resulted in higher values of TAC observed in both breast and thigh meat samples, as well as in liver tissue. These results underscore the antioxidative properties of pomegranate and onion extract, which hold the potential to enhance the preservation of meat product quality and freshness.

In conclusion, our results suggest that dietary interventions with pomegranate and onion extracts have the potential to influence the physiological status of chickens, with implications for their overall oxidative status. However, further research and exploration are necessary to gain a comprehensive understanding of the underlying mechanisms driving these antioxidative effects and to optimize their application in poultry nutrition, as the cyclodextrin extract of pomegranate and onion seemed to lower lipid oxidation in comparison to the aqueous one, whereas the aqueous extract of pomegranate and onion seemed to lower protein carbonyl formation and increased TAC in higher levels in comparison to the cyclodextrin one. Finally, dietary mixtures of herbal extracts with pomegranate and onion enhanced the hepatic energy status and exerted ameliorative effects on stress-related proteins. Additionally, these mixtures affected gene expression of methionine synthase and methionine sulfoxide reductase in the liver.

## Figures and Tables

**Table 1:** Genes and primer sequences used to amplify the genomic poultry DNA, amplicon size, and GenBank accession numbers.

Gene	Primer Pair Sequence	Amplicon Size	GenBank
MTR	5′-tatgctgctgtcaggtctgg-3′ 5′-tggctacagtcagggcttct-3′	146 bp	NM_001031104.1
TAT	5′-gctggagccatgtacctgat-3′ 5′-accacacggaagaagtttgg-3′	152 bp	XM_414240.5
SMS	5′-ctgcggttgattcttgacct-3′ 5′-atgtaggagggaacgcacac-3′	173 bp	NM_001030803.1
MSRB1	5′-gaggcgaagtgttcaaggac-3′ 5′-acttgccacaggacaccttt-3′	192 bp	NM_001135558.2
BHMT	5′-ggtgcttccattgttggagt-3′ 5′-caggtgggctttcagcttag-3′	108 bp	XM_414685.5
β-actin	5′-ctccctgatggtcaggtcat-3′ 5′-atgccagggtacattgtggt-3′	203 bp	L08165

MTR: methionine synthase, TAT: tyrosine aminotransferase, SMS: spermine synthase, MSRB1: methionine sulfoxide reductase B1, BHMT: betaine homocysteine S-methyltransferase.

**Table 2:** Effect of dietary supplementation with pomegranate and onion on broiler chicken breast, thigh, and liver values of TBARS ^1^, protein carbonyls, and TAC ^2^.

	Control ^3^	POMALAQ ^3^	POMALCD ^3^	SEM ^4^	*p*
Breast meat TBARS after iron oxidation (ng/g)					
Minute 30	4.95 ^a^	2.75 ^b^	1.45 ^c^	0.06	<0.001
Minute 60	12.8 ^a^	5.21 ^b^	4.95 ^b^	3.18	<0.001
Thigh meat TBARS after iron oxidation (ng/g)					
Minute 30	7.27 ^a^	5.77 ^b^	4.12 ^c^	0.12	<0.001
Minute 60	28.6 ^a^	16.5 ^b^	11.4 ^c^	0.18	<0.001
Liver TBARS after iron oxidation (ng/g)					
Minute 30	7.27 ^a^	5.77 ^b^	4.12 ^c^	0.12	<0.001
Minute 60	28.6 ^a^	16.5 ^b^	11.4 ^c^	0.18	<0.001
Protein carbonyls in breast meat after iron oxidation (ng/g)	8.33 ^a^	1.47 ^c^	2.99 ^b^	0.21	<0.001
Protein carbonyls in thigh meat after iron oxidation (ng/g)	9.87 ^a^	2.06 ^b^	3.75 ^b^	0.14	<0.001
Protein carbonyls in liver after iron oxidation (ng/g)	10.71 ^a^	3.31 ^b^	5.19 ^b^	0.28	<0.001
Breast meat TAC	14.1 ^c^	31.4 ^a^	22.9 ^b^	1.66	<0.001
Thigh meat TAC	25.7 ^c^	42.1 ^a^	33.5 ^b^	2.12	<0.001
Liver TAC	28.1 ^c^	52.6 ^a^	41.3 ^b^	3.31	<0.001

^1^ TBARS: thiobarbituric acid reactive substances; ^2^ TAC: total antioxidant capacity; ^3^ control, basal diet; POMALAQ, diet supplemented with aqueous pomegranate and onion extract at 0.1% per kg of DM; POMALCD, diet supplemented with pomegranate and onion in cyclodextrin at 0.1% per kg of DM; ^4^ SEM: standard error of mean; ^a–c^ values in the same row with no common superscript differ significantly (*p* < 0.05).

## Data Availability

The data presented in this study are available on request from the corresponding author.
